# Coupled FE Modeling of Superelastic NiTi Arch Wire and Bone Remodeling to Simulate 3D Orthodontic Tooth Movement During Leveling Phase

**DOI:** 10.1002/cnm.70217

**Published:** 2026-09-29

**Authors:** Mouhamad Abou Hamdan, Delphine Wagner, Hamdi Jmal, Siham Touchal

**Affiliations:** ^1^ Département Mécanique Université de Strasbourg, CNRS, ICube, UMR 7357 Strasbourg France; ^2^ Faculté de Chirurgie Dentaire Université de Strasbourg Strasbourg France; ^3^ Département d'Orthopédie Dento‐Faciale Les Hôpitaux Universitaires de Strasbourg Strasbourg France

**Keywords:** alveolar bone remodeling, dental biomechanics, finite element method, orthodontic tooth movement, periodontal ligament, superelastic behavior

## Abstract

Orthodontic tooth movement results from alveolar bone remodeling induced by mechanical loads applied to the teeth over extended periods during the unloading phase of arch wires. In clinical practice, orthodontic arch wires are inserted into bracket slots without precise knowledge of the forces transmitted to individual teeth. Excessive loads may lead to undesirable side effects, whereas insufficient forces may fail to initiate bone remodeling and tooth movement. This study aims to develop a 3D numerical approach based on finite element method to simulate orthodontic tooth movement during the alignment phase while quantifying the loads applied by the superelastic nickel titanium arch wire. A common clinical case of buccal infraposition of the maxillary canine was selected. Patient‐specific geometries of the teeth and alveolar bone were reconstructed from cone‐beam computed tomography scans. A coupling strategy between the arch wire unloading response and an external bone remodeling model was implemented to predict teeth movement over a one‐month treatment period as orthodontic appointments are typically scheduled every 4 weeks. During recovery to its initial shape, the arch wire delivered light and constant forces, leading to extrusion of the canine and intrusion of the adjacent first premolar and lateral incisor. This numerical framework captured the mechanical behavior of the superelastic nickel titanium arch wire and predicted teeth movement consistent with clinical ranges reported in the literature. This approach provides an efficient tool for orthodontists to predict patient‐specific orthodontic outcomes and optimize treatment design during the leveling phase.

## Graphical abstract

A 3D FE approach couples superelastic NiTi archwire unloading with alveolar bone remodeling to predict leveling‐phase orthodontic tooth movement. It quantifies the loads applied on individual teeth for controlled tooth movement and is applied to patient‐specific infra‐positioned canine alignment, predicting partial arch leveling with canine extrusion and adjacent teeth intrusion.

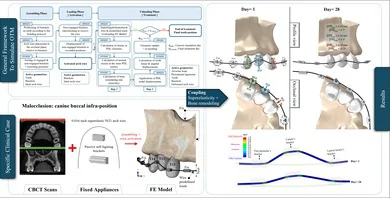

## 1 Introduction

Orthodontic treatment is required to correct different cases of dental malocclusions. Orthodontists propose a treatment plan based on their expertise trying to reach the ideal occlusion using appropriate orthodontic devices. During the initial phase of the treatment, superelastic arch wires made of Nickel Titanium (NiTi) alloys are widely used as an initial wire because of their unique biomedical material properties, which enable the delivery of light and nearly constant forces for long periods [1, 2, 3]. It has been shown that during activation and deactivation, the load‐deflection curves exhibit two plateaus revealing the forward and reverse martensitic transformations [2, 4, 5]. Orthodontists are primarily interested in the force plateau exhibited during the deactivation phase of superelastic arch wires [6]. This plateau corresponds to the orthodontic load applied to the teeth over an extended period as the wire gradually recovers its ideal arch form. Such sustained loading promotes alveolar bone remodeling by triggering cellular activity, with osteoclast‐mediated bone resorption on the compression side and osteoblast‐driven bone formation on the tensile side [7, 8, 9].

However, the treatment is considered effective when adequate mechanical loads are applied to misaligned teeth [10] producing controlled tooth movement while minimizing adverse effects such as root resorption, Periodontal Ligament (PDL) hyalinization, sterile necrosis, excessive inflammatory signaling [11, 12, 13] and minimum reaction forces on adjacent teeth. Consequently, considerable research efforts have focused on quantifying the loads applied by the arch wire on teeth. Analytical approaches have demonstrated that, when a continuous arch wire is engaged in multiple brackets across the entire dentition, the resulting force system becomes statically indeterminate, as it cannot be fully resolved using classical equilibrium equations [14, 15]. To overcome this limitation, some researchers relied on experimental measurement using load cells to quantify the orthodontic forces under various conditions [16, 17]. Others employed Finite Element Method (FEM) to gain insight into the forces delivered to teeth immediately after wire activation [18, 19, 20].

On the other hand, FEM is used frequently to simulate orthodontic bone remodeling, and existing predictive models can generally be divided into two main categories. The first category includes internal remodeling models, which incorporate the mechanobiology within the bone‐PDL complex without simulating the long‐term Orthodontic Tooth Movement (OTM). Within this framework, two main schools of thought have emerged, both centered on the identification of mechano‐sensors initiating bone remodeling during OTM. Some studies are based on the mechano‐stat theory derived from Wolff's law, whereby bone adapts to maintain optimal strain levels. In this context, osteocytes embedded within the bone matrices are assumed to sense the mechanical stimulus generated by orthodontic appliances and regulate alveolar bone density accordingly [10, 21, 22]. Other studies emphasize the bone‐PDL interface, proposing that fibroblasts within the PDL act as the primary mechano‐sensors driving orthodontic bone remodeling, thereby challenging the hypothesis that osteocytes alone can detect the relatively low deformations induced by orthodontic loading. Within this perspective, Van Schepdael et al. proposed a fully internal remodeling process integrating cellular dynamics and molecular signaling pathways activated by fibroblast mechano‐sensation in the PDL [23]. Similarly, Wagner et al. and Spingarn et al. introduced predictive models in which alveolar bone remodeling is governed by oxygen concentration and diffusion within the PDL [8, 24].

In contrast, external bone remodeling models rely on empirical relationships to simulate changes in tooth position and alveolar bone morphology without explicitly modeling the underlying cellular mechanisms of bone resorption and apposition. In these approaches, the material properties of all components are generally assumed to be constant, and the models are primarily designed to simulate long‐term OTM by focusing on global tooth displacement and treatment planning optimization. Most studies within this category consider the PDL as the biological driver of bone remodeling and subsequent tooth movement. Some approaches iteratively update tooth displacement based on initial PDL deformation induced by applied virtual forces to simulate space closure until teeth contact is achieved [25, 26], while others establish a relationship between the normal strains on the outer surface of the PDL and the remodeling rate derived from clinical data [27, 28, 29]. Alternatively, hydrostatic pressure within the PDL has also been proposed as a principal mechanical stimulus governing bone remodeling and subsequent teeth movement [30].

Owing to the inherent complexity of orthodontic mechanics, the previously mentioned FEM studies adopted simplified approaches. Some investigations focused exclusively on the initial loading state of the arch wire to quantify orthodontic loads immediately after activation, with subsequent OTM inferred from the resulting initial displacements of teeth, without explicitly simulating the deactivation phase of the wire. Other studies simulated OTM independently of treatment time, using the arch wire primarily as a kinematic guide to prescribe teeth displacement rather than as the mechanical component responsible for generating orthodontic loads. Additional approaches modeled OTM using simplified geometries and loading conditions, in which constant or time‐dependent decaying forces were applied to a single tooth crown to approximate the loads delivered by superelastic arch wires. In contrast, the aim of the present study is to develop an advanced 3D FEM‐based numerical method using patient‐specific geometries to simulate long‐term OTM during the alignment phase while quantifying the loads applied to individual teeth by the activated ideal arch form of a superelastic NiTi wire. This method couples the superelastic behavior of the arch wire and the resulting external bone remodeling inducing teeth movements.

## 2 Methods

This section presents the general 3D numerical framework developed to simulate OTM specifically under fixed orthodontic appliances. After introducing the methodology, it is applied to a specific clinical case involving a buccally displaced and infra‐positioned upper right canine treated using an initial superelastic NiTi arch wire. The model reproduces the clinical orthodontic workflow by first assembling the fixed appliances on misaligned teeth, followed by arch wire activation and the subsequent unloading phase representing the orthodontic treatment, where external bone remodeling is coupled with PDL mechanics to update teeth positions over the treatment period.

### 2.1 Orthodontic Tooth Movement Approach

Unlike simplified methodologies commonly reported in the literature, the present model is initialized from a patient‐specific malocclusion, providing a realistic and clinically relevant geometric configuration. An ideal arch form is then assembled to define the curvature of the orthodontic wire prior to activation. Rather than prescribing isolated or decaying forces on individual teeth, the wire is activated and subsequently allowed to release its stored stresses, generating a force system that acts on the multi‐tooth assembly. In addition, the loads transmitted to the teeth are quantified throughout the unloading phase of the wire, enabling continuous evaluation of the biomechanical response during treatment rather than limiting the analysis to the immediate post‐activation state. The complete numerical pipeline is summarized in Figure 1, and the different steps are described in further sections.

**FIGURE 1:**
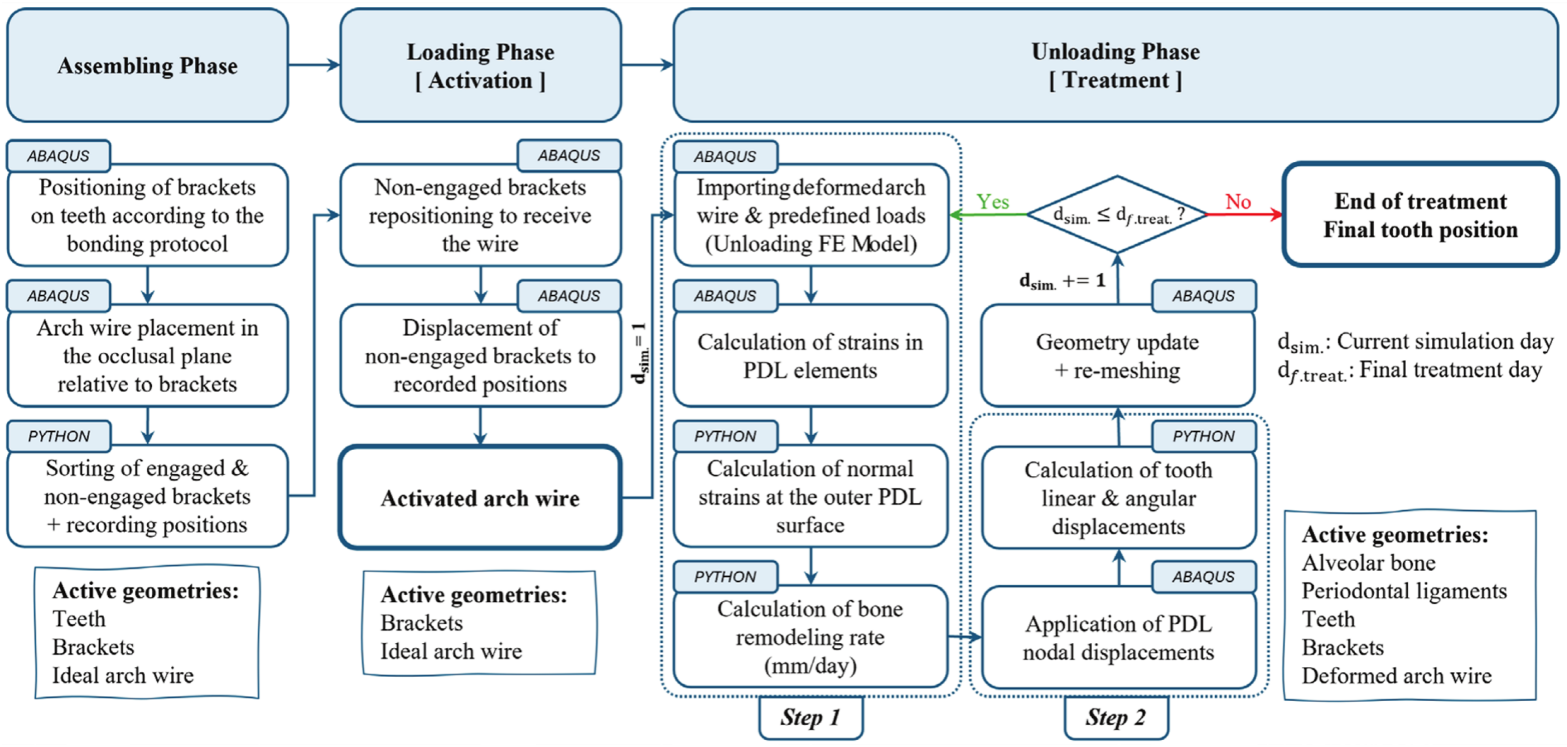
Flow chart describing the general 3D patient‐specific orthodontic tooth movement approach.

#### 2.1.1 Assembling Phase

Consistent with standard clinical practice, the modeled brackets, regardless of their design or prescription, are positioned on the crowns of all teeth following the established bonding protocol. The arch wire, modeled in its ideal arch form, is then placed in the occlusal plane relative to the bracket slots while maintaining coincidence between the arch wire midline and the dental midline. Subsequently, the teeth are suppressed from the model, as they serve solely to establish the precise spatial positioning of the brackets.

For brackets located outside the ideal arch form or displaced from the occlusal plane, the arch wire cannot be directly inserted into their slots because the bracket slots either do not align with the wire trajectory or geometrically intersect it. These brackets are identified as non‐engaged brackets requiring wire deflection for engagement. Their positions and orientations on the misaligned teeth are recorded to serve as target values during the loading phase.

#### 2.1.2 Loading or Activation Phase

Before activating the arch wire, it is necessary to ensure that it is fully engaged within all bracket slots. To achieve this, the non‐engaged brackets are repositioned so that the wire fits inside their slots without initial geometric interpenetration. Therefore, prescribed translational and rotational displacements are then applied to those brackets to return them to the positions and orientations recorded on the misaligned teeth during the assembling phase. Throughout this step, the initially engaged brackets remain fixed in all degrees of freedom.

Although this numerical strategy does not exactly replicate the clinical insertion technique, during which the clinicians typically bend the arch wire during placement, it reproduces the biomechanically equivalent activated state of the arch wire. This includes the generated internal stresses, deformations, and reaction forces during engagement. The resulting activated configuration then serves as the initial state for the subsequent treatment phase.

#### 2.1.3 Unloading or Treatment Phase

The unloading phase represents the actual progression of orthodontic treatment, during which the arch wire releases forces and the bone remodeling progresses around the teeth. This process causes teeth movement and progressive sliding of the arch wire until it recovers its initial ideal shape.

The first step involves releasing the arch wire, allowing it to exert forces on the teeth. For this purpose, on the first day of treatment, the deformed configuration of the arch wire with its predefined loads obtained at the end of the activation phase, is imported into a separate unloading FE model. This model includes the teeth, their corresponding PDLs, the bonded brackets, and the alveolar bone. The forces released by the activated arch wire produce an initial displacement of the teeth by deforming their surrounding PDLs. The normal strains $\varepsilon_{n}$ generated within the PDL were used as the mechanical stimulus driving alveolar bone remodeling [27], consistent with previous studies showing that normal strain‐based stimuli enable accurate prediction of clinically relevant OTM [27, 28, 29]. Based on this concept, the empirical relationship in Equation (1) was established between the normal strains at the outer surface of the PDL and the orthodontic bone remodeling rate $\dot{R}$. A minimum strain threshold of 0.03% was assumed to initiate bone remodeling. Within the range of 0.03% to 0.3%, the alveolar bone remodeling rate was considered to increase linearly with the strain experienced in the PDL, reaching a maximum velocity of approximately 0.0414 mm/day.

(1) $$\dot{R}\left(\mathrm{mm}/\mathrm{day}\right)=\left\{\begin{array}{l} \;0\\ \;0.153\;\varepsilon_{n}-0.0046\\ \;0.0414 \end{array}\right.\;\;\;\begin{array}{c} \varepsilon_{n}<0.0003\\ 0.0003<\varepsilon_{n}<0.003\\ \varepsilon_{n}>0.003 \end{array}$$

Different scenarios were evaluated to determine the most appropriate direction of the remodeling vector. The most consistent results were obtained when the remodeling direction was defined by normalizing the displacement components of each node along the three axes by the corresponding maximum displacement magnitude as expressed in Equation (2).

(2) $$\vec{U}=\left\{\;\frac{U_{x}^{\max}}{\left\|{\vec{U}}^{\max}\right\|};\frac{U_{y}^{\max}}{\left\|{\vec{U}}^{\max}\right\|};\frac{U_{z}^{\max}}{\left\|{\vec{U}}^{\max}\right\|}\;\right\}$$

The second step simulated bone remodeling by imposing the calculated nodal incremental displacements $\vec{\dot{R}}=\dot{R}\times \vec{U}$ on the outer PDLs surfaces. This stage enabled the quantification of the resulting daily tooth movement.

At the beginning of each first unloading step, the geometries of teeth, PDLs, bone, and brackets are updated. The bone and PDLs are then re‐meshed, and the deformed arch wire carrying its predefined loads from the previous day is re‐imported to initialize the next treatment day.

Through the automated python scripts running inside ABAQUS, the iterative unloading phase can thus be simulated over any desired treatment duration.

### 2.2 Application on a Patient‐Specific Geometry

To find out the effectiveness of the proposed model, a patient‐specific clinical case presenting infraposition of the canine, a malocclusion frequently encountered in orthodontic practice, was selected. The model was then used to simulate OTM during the alignment phase for a 28‐day treatment period using an initial superelastic NiTi arch wire.

#### 2.2.1 Geometric Configuration

After post‐processing the Cone Beam Computed Tomography (CBCT) scans of an anonymized patient from Strasbourg Hospital, the geometries of all upper right teeth and the maxillary bone were reconstructed using 3D Slicer. Passive self‐ligating brackets from the Victory Series, with nominal slot dimensions of 0.018 × 0.025‐in. were geometrically simplified and modeled according to their nominal dimensions. For the first molar, the orthodontic attachment was modeled as a buccal tube with an internal diameter of 0.018‐in. Within this appliance configuration, a round 0.016‐in. BROAD ARCH Upper Large arch wire from Ormco was modeled using ABAQUS. In the rectangular bracket slots, the arch wire had a nominal total clearance of 0.002‐in. in the coronoapical direction and 0.009‐in. in the buccolingual direction. In the first molar buccal tube, the total diametral clearance was 0.002‐in. As illustrated in Figure 2, the present case is characterized by a buccal infraposition of the upper right canine of approximately 3 mm.

**FIGURE 2:**
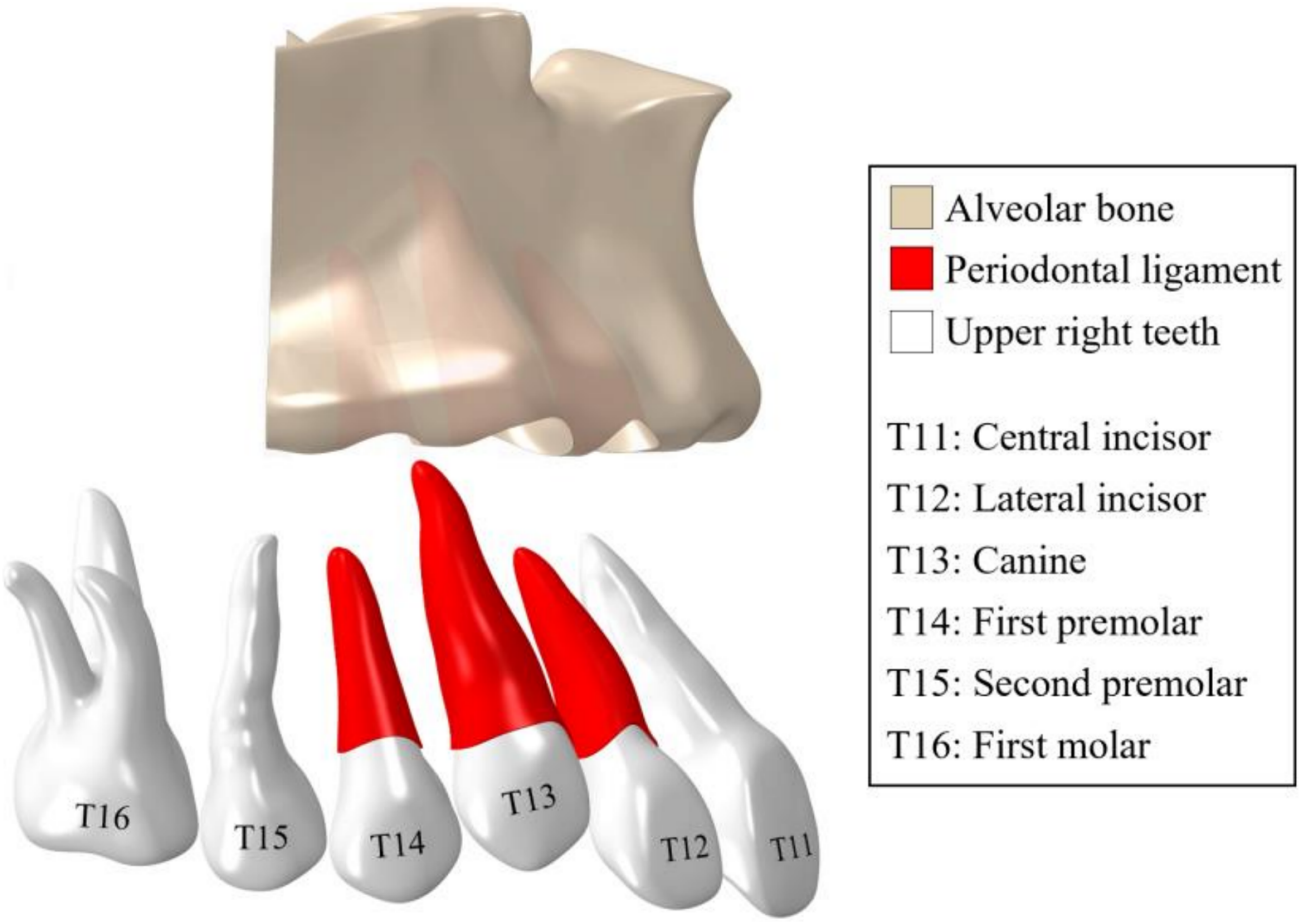
Initial 3D geometric configuration of the patient‐specific case in right profile view, showing the infrapositioned maxillary canine, the adjacent teeth of the upper right segment, the PDLs of the studied teeth, and the corresponding alveolar bone.

For simplification, only the movements of the upper right first premolar, canine, and lateral incisor are considered in this study. To improve the computational efficiency, their corresponding bone is cut from the full maxillary bone as shown in Figure 2, and the arch wire is cut at its midline and from the distal side as shown in Figure 3 and the PDLs for those teeth are expended from the teeth root surfaces with a uniform thickness of 0.2 mm [27, 31]. This interpretation is also supported by FE studies showing that modeling the PDL with a uniform thickness yields results comparable to those obtained using realistic, CT‐derived thickness distributions, with only minimal differences in tooth displacement patterns and center of resistance [32]. Afterwards, teeth and PDLs are differentiated from bone geometry.

**FIGURE 3:**
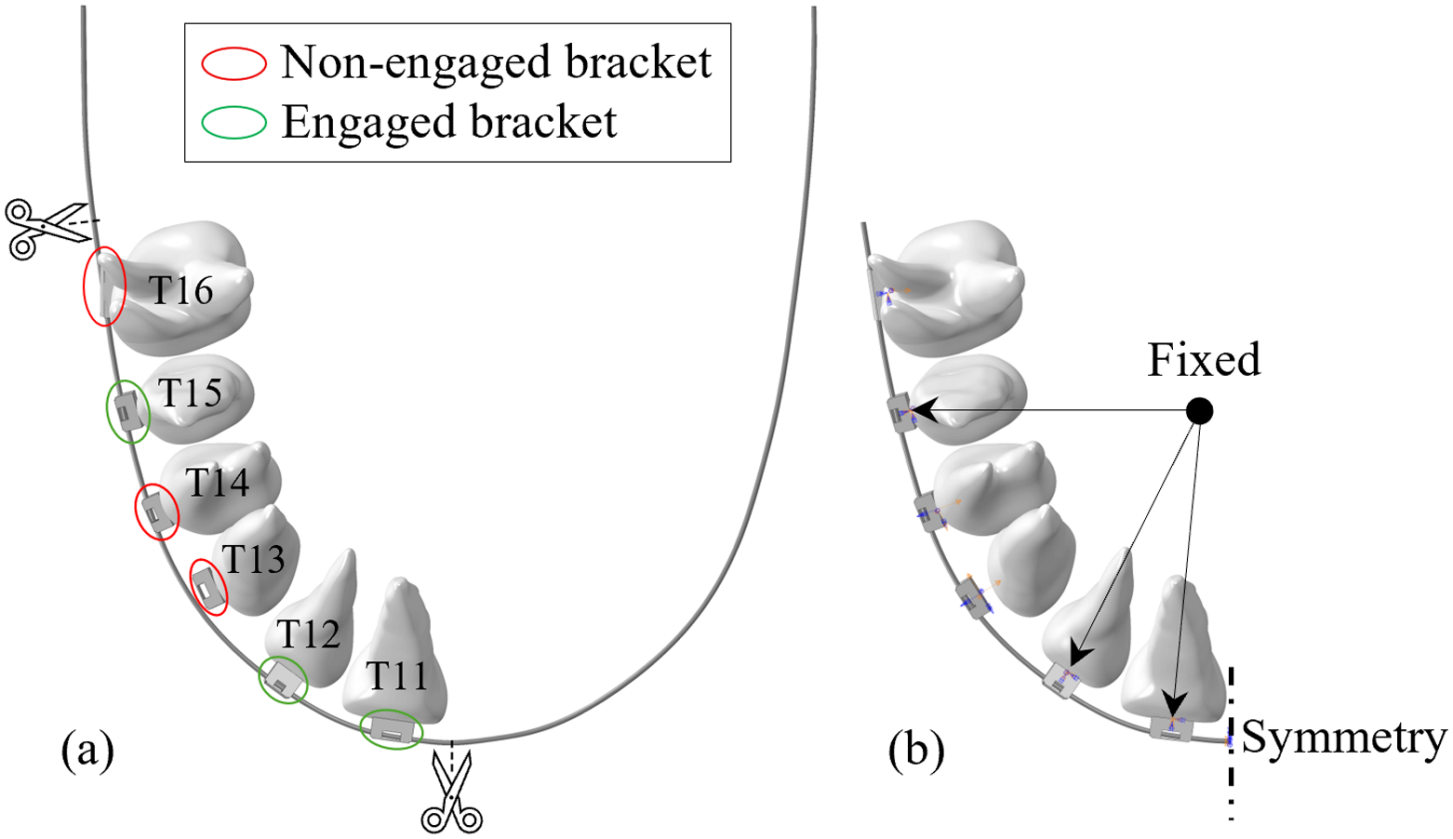
Top view of the 3D model during the assembling and activation phases. (a) Ideal 0.016‐in. arch wire placed in the occlusal plane relative to the brackets positioned on teeth, with engaged and non‐engaged brackets indicated by green and red circles, respectively. (b) Simplified activation model after repositioning of the non‐engaged brackets to receive the wire. The teeth are then suppressed and excluded from the loading simulation.

At first, the brackets are bonded to the crowns of misaligned teeth, and the arch wire is placed in the occlusal plane relative to the brackets while maintaining proper midline alignment (Figure 3a). In this step, the brackets associated with T11, T12, and T15 were properly engaged. However, geometric interpenetration occurred between the wire and the brackets associated with T14 and T16, while the bracket corresponding to T13 was outside the wire trajectory. These brackets are classified as non‐engaged, and their positions and orientations are recorded. They are then repositioned to receive the wire (Figure 3b). During the activation simulation, translational and rotational displacements are applied to the non‐engaged brackets to return them to their recorded positions and orientations on the misaligned teeth, thereby deflecting and activating the arch wire. Afterwards, the treatment duration is specified for one month because orthodontic appointments are typically scheduled every 4 weeks.

#### 2.2.2 Material Properties

The alveolar bone was modeled without separating cortical and cancellous regions to improve computational efficiency, and because this distinction has only a minor effect on the FE results. Instead, it was represented as a homogeneous, isotropic, and linear elastic material as done in literature [8, 28, 29, 33, 34]. Despite the PDL's inherently nonlinear and viscoelastic behavior, numerous studies have continued to represent it using linear elastic properties [22, 27, 28, 29]. Moreover, some researchers demonstrated that accounting for PDL nonlinearity has minimal influence on both the strain distribution within the ligament and the resulting tooth displacement patterns [32, 35]. Teeth were assumed as rigid bodies because their stiffness is markedly greater than that of the PDLs. The passive self‐ligating brackets were considered also as rigid bodies, as stainless steel exhibits a substantially higher elastic modulus than NiTi, making bracket deformation negligible under the applied loading conditions. To model the mechanical behavior of the shape memory alloys, the superelastic constitutive law in ABAQUS that is based on the work of Auricchio and Taylor [36, 37] is used for its simplicity rather than other complex models incorporating the reorientation of martensite under thermomechanical loadings. This model was selected over other available macroscale constitutive formulations because it demonstrates strong agreement with experimental data, particularly in bending‐type deformation scenarios [4, 38, 39]. Both martensite and austenite phases are represented using isotropic linear elasticity. During the phase transformation, the effective elastic properties are computed as a weighted combination of the austenite and martensite elastic constants, following the law of mixtures. At constant temperature near the austenite finish temperature and without loading, the superelastic material is assumed in the austenite phase and furthermore after loading, a forward martensitic transformation will occur transforming it to martensite controlled by the current stress state. Specifically for superelastic NiTi wires, the model parameters identified through experimental uniaxial tensile tests performed at oral temperature 36°C ± 1°C are used from [4] and reported in Table 1 along with the material properties used for PDL and alveolar bone from [29]. Although oral temperature may vary with food intake and ambient air exposure, the temperature was assumed to remain constant throughout the treatment period.

**TABLE 1:** Material properties used in the FE model.

Part	Constitutive behavior	Parameters
PDL	Isotropic linear elastic	E = 0.68 MPa v = 0.49
Bone	Isotropic linear elastic	E = 20 GPa v = 0.3
Arch wire	Isotropic superelastic (Auricchio Model)	Elastic parameters $E_{A}=48.7\;\mathrm{GPa}\;|\;v_{A}=0.33$ $E_{M}=19\;\mathrm{GPa}\;|\;v_{M}=0.3$ $\varepsilon_{L}=0.0335$ Forward transformation (Loading) $\sigma_{t}^{S}=385.66\;\mathrm{MPa}=\sigma_{c}^{S}$ $\sigma_{t}^{E}=399.20\;\mathrm{MPa}$ Reverse transformation (Unloading) $\sigma_{t}^{S}=120.18\;\mathrm{MPa}$ $\sigma_{t}^{E}=101.82\;\mathrm{MPa}$

In the superelastic model, the subscripts *A* and *M* denote the austenitic and martensitic phases, respectively. The parameter $\varepsilon_{L}$ represents the transformation strain. The symbol σ corresponds to the transformation stress in loading and unloading, with superscripts *S* and *E* indicating the start and the end of the transformation, respectively. While the subscripts *t* and *c* refer to the tensile and compressive states, respectively.

The partition technique was used to divide the PDLs, teeth, and the inner surface of the maxillary bone that is in contact with the outer surface of the PDL on one level, allowing for finer elements at their very curved apical zone reproducing their geometric configuration. A mesh convergence analysis was conducted based on the maximum normal strain at the outer surface of the PDLs, which represents the mechanical stimulus driving bone remodeling. This analysis was performed to determine an appropriate element size within the complex bone‐PDL‐tooth interface. In addition, a sufficiently refined mesh was adopted in the wire‐bracket complex to ensure accurate contact interaction and reliable force transmission. The boundary conditions correspond to the release of the arch wire following activation on the first day of the treatment. Based on the convergence results, an element size of 0.3 mm was adopted along the coronal region of the alveolar bone, while a refined size equal to half this value was assigned to the highly curved apical region. With this discretization, the estimated numerical error at the PDL‐tooth‐bone interfaces remained below 0.31% for the first premolar and 1.86% for the canine. A slightly higher error of 3.17% was observed in the region adjacent to the lateral incisor; this level was considered as a compromise between solution accuracy and computational efficiency. The final FE model consists of 769,845 elements in total, and Table 2 shows the number and types of elements used for each component.

**TABLE 2:** Mesh characteristics used in the FE model.

Part	Number of elements	Type
PDLs	120,040	C3D4
Bone	563,214	C3D4
Arch wire	27,186	C3D20R
Teeth	42,678	R3D3
Brackets	16,727	R3D4

#### 2.2.3 Interactions and Boundary Conditions

In the activation simulation, only the brackets and the arch wire were considered in the FE model. Therefore, surface‐to‐surface contact was defined between the wire and each bracket slot with a friction coefficient of 0.2 [40]. The engaged brackets are fixed in all degrees of freedom, whereas the non‐engaged brackets are displaced while maintaining a symmetry constraint at the arch wire midline.

In the treatment simulations, the complex bone‐PDL‐tooth was tied. The brackets were assumed to be perfectly bonded to their corresponding teeth. A kinematic coupling constraint was defined between the reference point of each rigid bracket and the reference point of its corresponding rigid tooth. All six degrees of freedom were constrained, ensuring that the bracket and tooth moved together without relative translation or rotation. In the first treatment day, the deformed configuration and internal stress state of the arch wire at the final frame of the activation simulation were imported into the unloading FE model (Figure 4). The same contact interaction is assigned in the brackets‐wire complex as in the activation simulation. The mesial and distal bone surfaces and the brackets corresponding to T11, T15, and T16 were considered fixed while applying a symmetry constraint at the arch wire midline.

**FIGURE 4:**
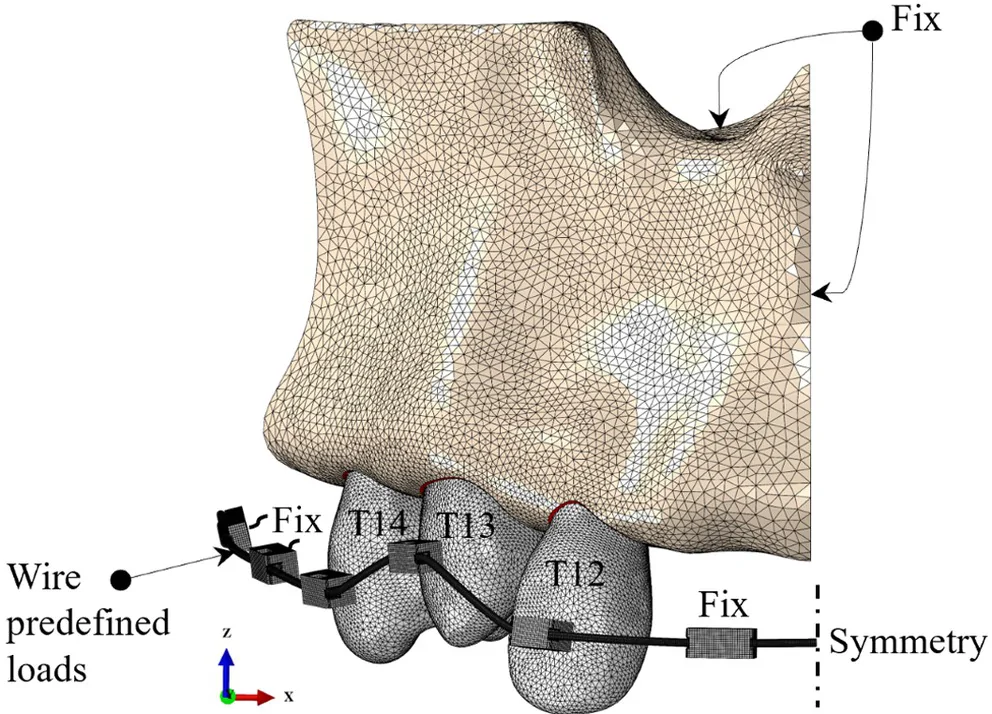
Boundary conditions applied to the unloading FE model during the treatment phase (front view).

## 3 Results

During the loading phase, as the malocclusion was mainly concentrated on the intrusion of the upper right canine, the bracket associated with the canine experienced the highest force magnitude, reaching 9.10 N after reaching its intended position on the canine crown (Figure 5). The brackets corresponding to lateral incisor and first premolar exhibited lower force levels, reaching 5.61 and 3.65 N, respectively at the end of the activation phase. The stress evolution along the arch wire was consistent with the martensitic transformation characteristics of NiTi.

**FIGURE 5:**
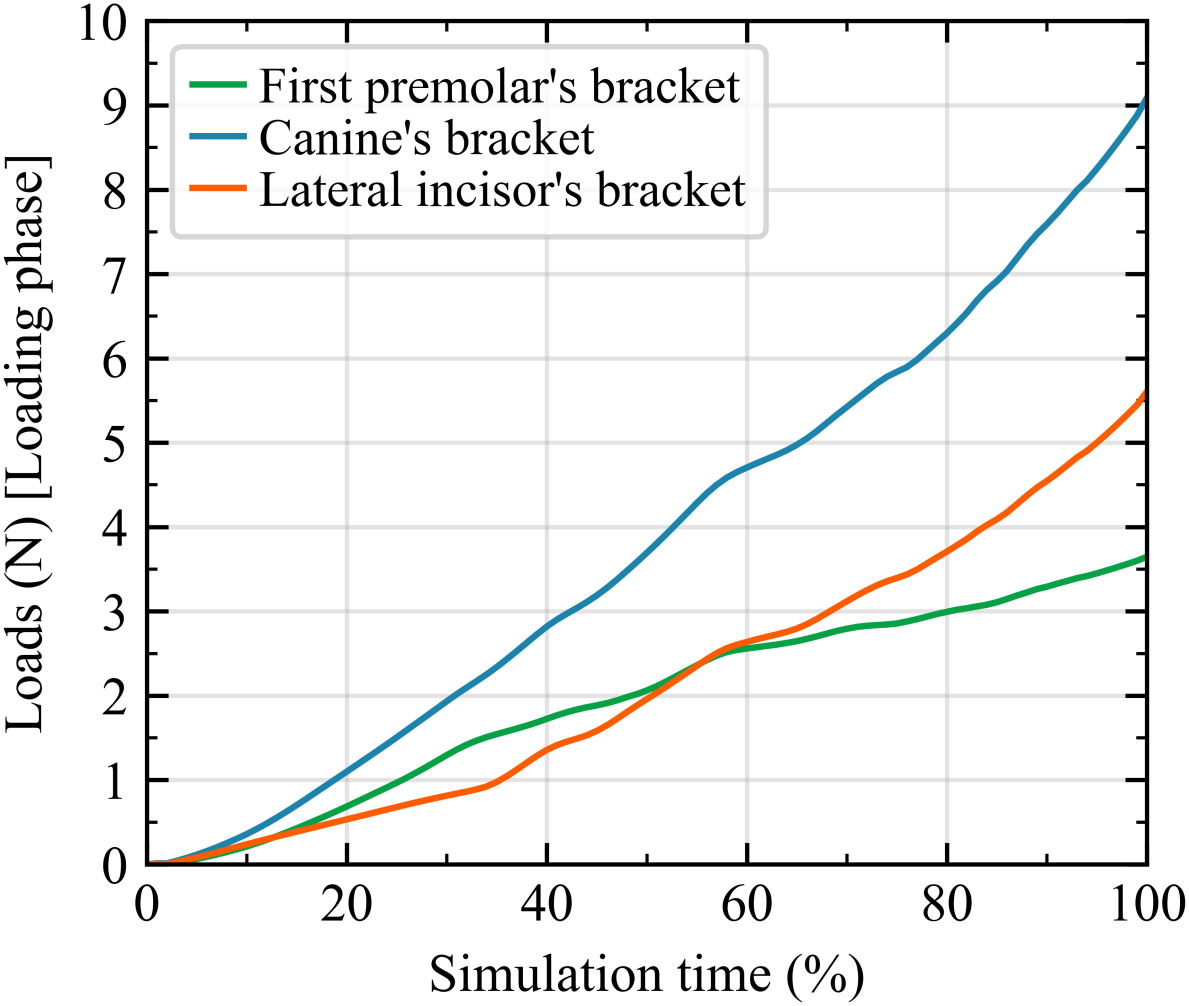
Applied loads on brackets during the activation phase of the wire.

During the unloading phase, the stresses stored in the wire during activation were progressively released, generating the forces responsible for teeth movement and alveolar bone remodeling. As observed in Figure 6, immediately after unloading, all brackets experienced a sharp reduction in load as the wire began recovering its original shape by the end of the first day. Subsequently, the loads continued to decrease more gradually and reached a quasi‐stabilized level after the tenth day. During this stabilized phase, the canine consistently exhibited the highest average load (0.82 N), followed by the first premolar (0.48 N), while the lateral incisor experienced the lowest loads (0.21 N).

**FIGURE 6:**
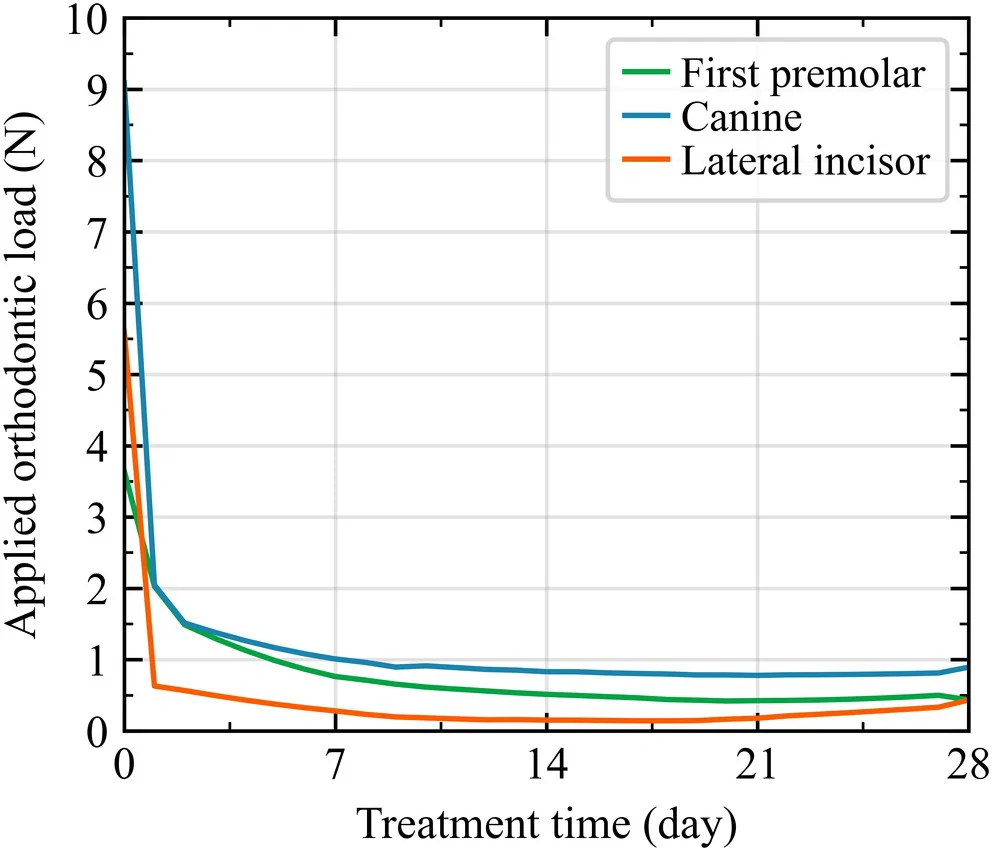
Applied orthodontic loads on teeth during the 28‐day treatment period.

Teeth movement was quantified using a rigid‐body kinematic description. For each specific tooth, an anatomical tooth coordinate system was defined in the initial configuration (Figure 7), with the coronoapical axis aligned with the vector from the crown tip (point C) to the apex (point A), the mesiodistal axis defined from a mesial reference direction (point M) and orthogonalized to the coronoapical axis, and the buccolingual axis obtained by enforcing a right‐handed coordinate system.

**FIGURE 7:**
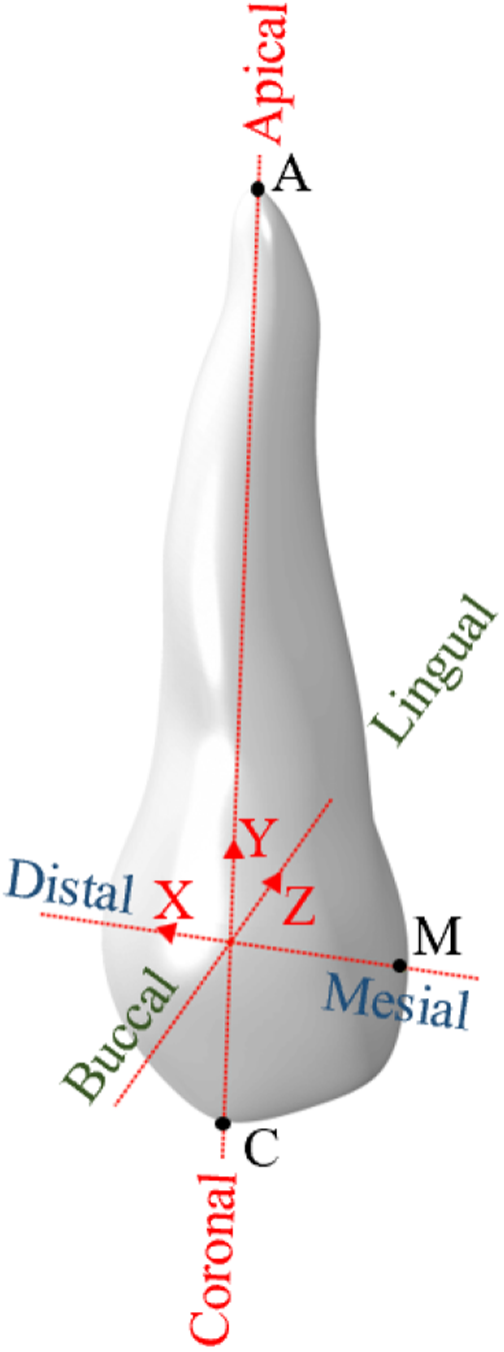
Initial anatomical coordinate system for the upper right canine, used to evaluate its movement.

The three non‐collinear anatomical landmarks were tracked during the unloading phase and the rigid‐body rotation between the initial and current configurations was computed from the relative change in the associated orthonormal triads, yielding a unique rotation matrix mapping the initial tooth orientation to the current one. The translational component of tooth movement was obtained from the displacement of a representative rigid point, defined as the centroid of the tracked landmarks, and was expressed in the initial anatomical coordinate system. Tooth orientation changes were then quantified by expressing the rigid‐body rotation in the same anatomical coordinate system and extracting the corresponding rotation angles about the mesiodistal, coronoapical, and buccolingual axes (Table 3). This will provide a clear clinical interpretation of the simulated tooth movement in terms of translation, tipping, and axial rotation.

**TABLE 3:** Apical, coronal and mesial landmarks displacements and the resulting translation and rotation of each tooth at the end of the treatment.

	First premolar				Canine				Lateral incisor			
	*X*	*Y*	*Z*	|Mag|	*X*	*Y*	*Z*	|Mag|	*X*	*Y*	*Z*	|Mag|
${\vec{U}}_{A}$ (mm)	−0.06	0.58	0.13	0.60	−0.04	−0.75	0.10	0.76	−0.11	0.16	0.11	0.22
${\vec{U}}_{C}$ (mm)	−0.08	0.58	0.20	0.62	−0.09	−0.74	−0.37	0.83	0.18	0.18	−1.01	1.04
${\vec{U}}_{M}$ (mm)	−0.02	0.62	0.05	0.62	−0.08	−0.74	−0.15	0.76	0.16	0.10	−0.04	0.19
$\vec{\mathrm{UT}}$ (mm)	−0.01	0.59	0.12	0.60	−0.07	−0.74	−0.14	0.76	0.08	0.15	−0.31	0.35
$\vec{\mathrm{UR}}$ (deg)	−0.35	−2.58	−0.67	2.69	1.11	1.77	−0.11	2.09	2.67	10.72	0.97	11.10

The results presented in Table 3 and Figure 8 indicate that partial recovery of the arch wire toward its original shape occurred during the simulation, leading to bone remodeling and inducing extrusion of the canine accompanied by intrusion of the adjacent teeth. The resulting teeth displacement can be visualized in Video S1.

**FIGURE 8:**
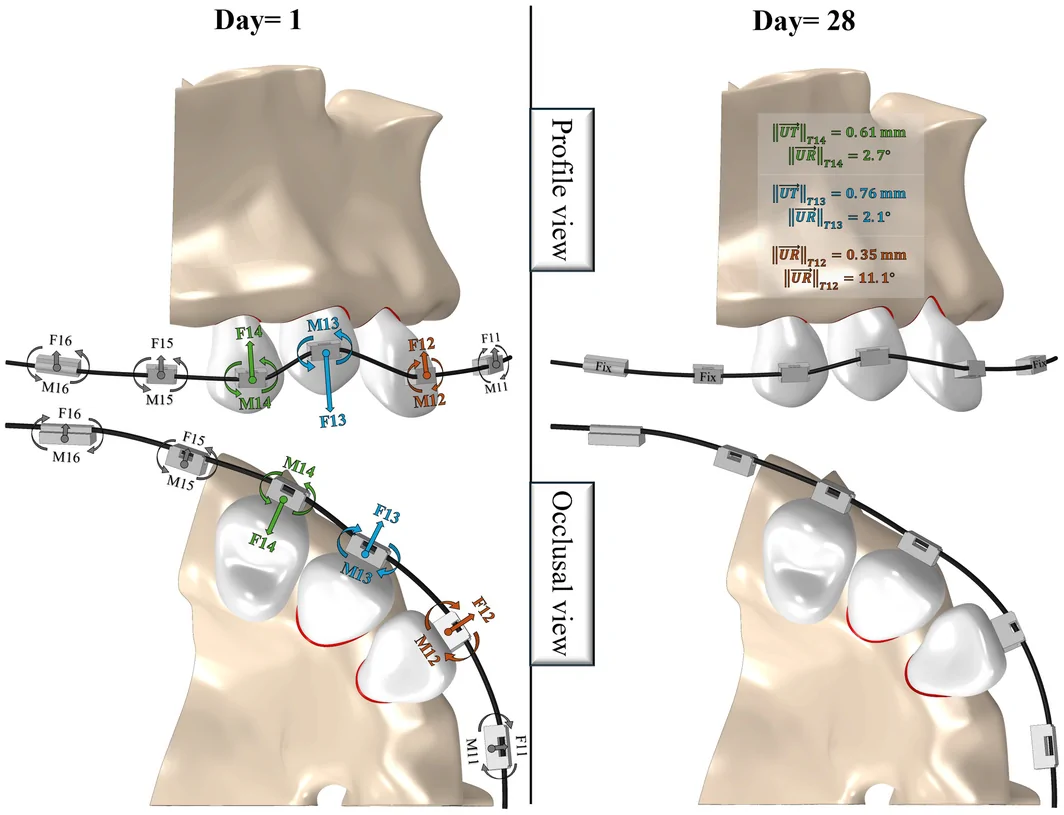
Initial and final configurations obtained during the long‐term bone remodeling simulation shown in profile and occlusal view. Forces and moments applied by the arch wire on the brackets are shown on the first day.

For the first premolar, the displacements of the apex, crown, and mesial landmarks exhibited consistent coronoapical motion, with values ranging from 0.58 to 0.62 mm. This coherent landmark behavior was reflected in a dominant coronoapical translational component corresponding to an intrusion of 0.59 mm, together with a smaller lingual translational component of 0.12 mm. Rotational components remained limited, with a total rotation magnitude of 2.69°, predominantly associated with axial rotation. These results indicate that first premolar movement was mainly characterized by pure translation with minor orientation changes.

For the canine, all landmarks showed similar apicocoronal displacement trends of approximately 0.74 mm, corresponding to an extrusion of 0.74 mm, together with a smaller buccal translational component of 0.14 mm. The apical landmark displaced in the lingual direction, whereas the coronal landmark moved buccally, indicating a buccolingual tipping of 1.11°. The magnitude of this rotation remained limited. In addition, an axial rotation of 1.77° about the coronoapical axis was observed. The total rotation magnitude reached 2.09°, indicating that rotational effects were present but small compared with the dominant translational motion.

The lateral incisor exhibited a markedly different movement pattern, characterized by limited translational motion and pronounced rotational responses. The coronoapical translational component corresponded to an intrusion of 0.15 mm, accompanied by a buccal displacement of approximately 0.31 mm. In contrast, the total rotation magnitude reached 11.10°, dominated by axial rotation about the coronoapical axis (10.72°) and accompanied by buccolingual tipping of 2.67°. These results indicate that lateral incisor movement was predominantly rotational, with comparatively limited translation.

Since the normal strains within the PDLs, induced by stress transfer from the arch wire, are considered the primary stimulus governing bone remodeling, the coronoapical normal strain distributions in the PDL of each tooth on the first day are presented in Figure 9. In the apical region, the canine exhibited important tensile strains, whereas the PDLs of the adjacent teeth predominantly experienced compressive strains. In particular, for the first premolar, additional compressive normal strains are observed in the furcation region.

**FIGURE 9:**
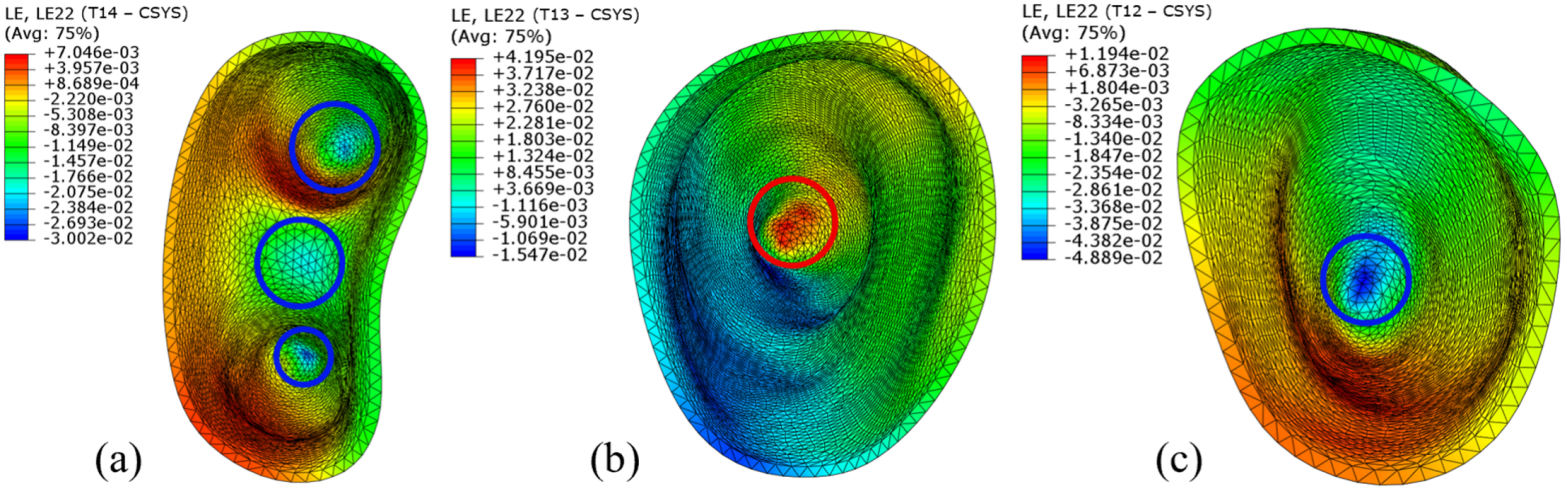
Occlusal view of different PDLs showing the logarithmic normal strain in the coronoapical direction of each tooth on the first day of the treatment: (a) First premolar; (b) Canine; (c) Lateral incisor.

Moreover, Figure 10 illustrates the time‐dependent evolution of coronoapical displacements expressed in the local coordinate system of each tooth throughout the treatment period. Distinct displacement rate patterns were observed among the three teeth. The lateral incisor exhibited an immediate decrease in translation rate from the first day, resulting in early stabilization with a negligible average coronoapical displacement rate of approximately 0.003 mm/day as the arch wire progressively recovered its shape. In contrast, the first premolar showed a gradual response, with an initial intrusion rate of approximately 0.036 mm/day in the first week, followed by a progressive decrease to an average rate of 0.016 mm/day. Similarly, the canine displayed the highest response, with an extrusion rate of approximately 0.042 mm/day during the first week, which then gradually decreased over time to an average rate of 0.019 mm/day for the remainder of the treatment.

**FIGURE 10:**
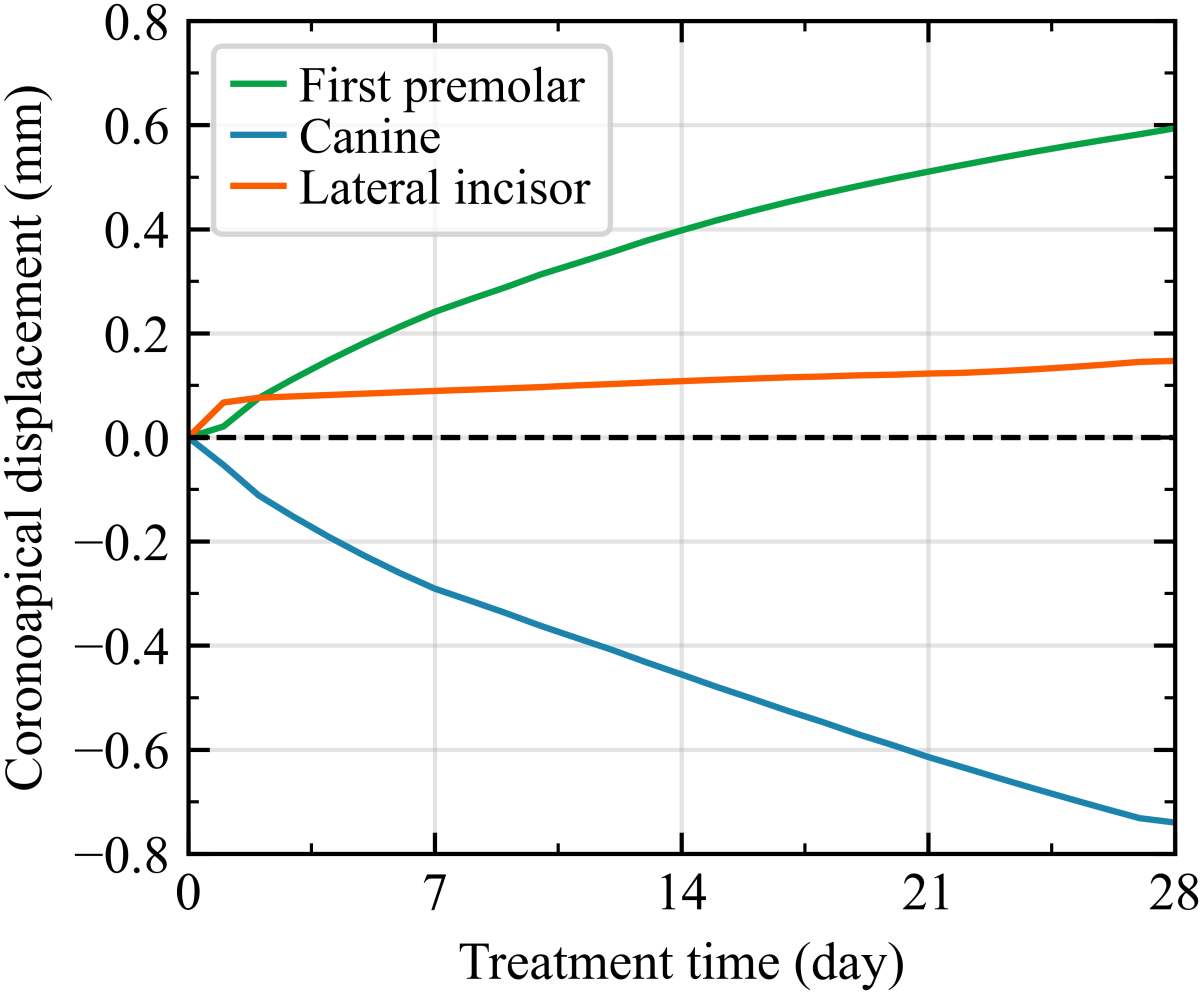
Evolution of coronoapical displacements presented in local coordinate systems of teeth over the treatment period.

## 4 Discussion

In this work, a 3D FEM approach was developed to simulate realistic OTM using the ideal arch form of the wire and brackets. This method is then applied to replicate the first phase of the orthodontic treatment called leveling phase. During this phase, practitioners very often use superelastic materials, such as NiTi arch wires, to achieve dental leveling. When the arch wire is inserted into the slots of brackets already bonded to misaligned teeth, the wire is effectively activated and subjected to mechanical loading. Under these conditions, the shape memory alloy arch wire undergoes a stress‐induced martensitic transformation. This response initially exhibits a linear elastic behavior characteristic of the austenitic phase, followed by a stress plateau during which austenite progressively transforms into martensite in the highly stressed regions, until the material becomes fully martensitic.

The malocclusion considered in this study is commonly encountered in orthodontic practice and consists of an infraposition of the right maxillary canine. This case was assumed to be treated using an initial 0.016‐in. superelastic NiTi arch wire. The simulation focused on the upper right dental segment, specifically the lateral incisor, canine, and first premolar. During the loading phase, the displacement imposed on the brackets induced localized bending of the wire, leading to an increase in stress in the most curved regions of the arch wire. As the local stress reaches the critical transformation stress defined in the constitutive model, a stress‐induced phase transformation from austenite to martensite was initiated, resulting initially in a partial transformation in the most stressed regions of the wire. As the wire deformation increased, the transformation evolved and some regions reached a fully martensitic state. By the end of the activation phase, the wire exhibited an extensive martensitic region, particularly localized at the most deflected portions of the wire in contact with brackets associated with T12, T13, and T14, corresponding to the regions experiencing the highest bending stresses.

Although the resultant force predicted at the end of the activation phase reached 9.10 N, this value should be interpreted as a transient engagement force rather than as the sustained orthodontic force applied during treatment. The coronoapical component of this extrusive force was 6.83 N for a prescribed coronoapical bracket displacement of 2.65 mm. This value is consistent with experimental measurements reported for superelastic NiTi arch wires under vertical activation. Wagner et al. [2] measured coronoapical forces of approximately 6 N at the same coronoapical displacement as in the present numerical study, using thinner 0.014‐in. NiTi arch wire with 0.018 × 0.025‐in. conventional brackets at 35°C. Although conventional ligation may increase the resistance to wire sliding and binding, the vertical displacement in their experimental setup was applied using a narrow loading tip. In contrast, in the present FE model, the wire was engaged inside the canine bracket slot, which had a mesiodistal width of 3.2 mm and was deformed by prescribed 3D translations and rotations. Therefore, in addition to the larger wire diameter, the different loading method and the mesiodistal width of the bracket may have increased the bending deformation and stresses in the arch wire. Similarly, Hsu et al. [41] reported high activation forces using H4 passive self‐ligating brackets with a larger 0.022 × 0.026‐in. slot. In their setup, the bracket was vertically displaced to deform the 0.014‐in. ThermoNiTi wire, generating approximately 7 N at the same vertical deflection. Therefore, although the numerical resultant force is high, its order of magnitude is supported by experimental activation tests. This is especially considering that the present model used a larger 0.016‐in. NiTi arch wire, a smaller 0.018 × 0.025‐in. passive self‐ligating bracket slot, and the wire deflection induced by the prescribed translations and rotations of the brackets.

At the beginning of the numerical treatment, the arch wire slides through the slots of the brackets as the teeth began to move, resulting in a rapid decay of the initial applied forces. As shown in Figure 6, this mechanical response produced a transition toward relatively light and stable forces for the majority of the treatment period, which is needed by the practitioners to prevent root resorption, PDL hyalinization, sterile necrosis and inflammatory signaling at heavy loads [11, 13, 42] and that is consistent with experimental observations for superelastic NiTi arch wires [2]. In the present simulation, the highest force magnitude was observed on the canine, reaching 83.6 g, which remained within the clinically acceptable range and below the upper threshold of 150 g reported in [43], whereas lower force magnitudes were predicted for the adjacent lateral incisor and first premolar.

During the first day of treatment, the coronoapical normal strain distributions within the PDLs depicted in Figure 9, revealed pronounced tensile strains in the apical region of the canine. These tensile regions correspond to zones of bone formation and are consistent with the early extrusion observed for this tooth. In contrast, compressive strains were predominantly observed in the apical regions of the lateral incisor and the first premolar, corresponding to bone resorption and the initial intrusion of these teeth. On this first day, compressive strains were higher in the PDL of the lateral incisor, which has a single root. Whereas in the PDL of the first premolar, the compressive strains were distributed between the two roots and the furcation region. This difference in strain distribution along with the lower applied forces initially may explain the lower coronoapical translational response of the first premolar compared with other teeth during the first day (Figure 10).

Over the 28‐day treatment period, the fully martensitic regions initially located in the highly bent portions of the arch wire progressively decreased as reverse transformation from martensite to austenite occurred, although localized martensitic zones remained present (Figure 11). This partial reverse transformation led to a redistribution of relatively light and sustained forces delivered by the arch wire. Consequently, the canine showed pronounced extrusion accompanied by limited axial rotation and buccolingual tipping, while the adjacent teeth underwent intrusion. The first premolar experienced a higher force plateau than the lateral incisor, which was associated with a greater intrusion rate. In contrast, for the lateral incisor, owing to the clearance between the arch wire and its bracket slot, the initial teeth displacement, particularly the extrusion of the canine, allowed the arch wire to slide buccally within the fixed bracket bonded to the central incisor and along the lower mesial wing of the lateral incisor's bracket. This sliding mechanism allowed the arch wire to accommodate the deformation and gain effective length, given that the wire midline was constrained. The application of these light forces generates a positive moment about the coronoapical, buccolingual, and mesiodistal axes. This mechanical environment explains the limited coronoapical translational movement of the lateral incisor, as well as the increased rotational response observed in Table 3 and Figure 10.

**FIGURE 11:**
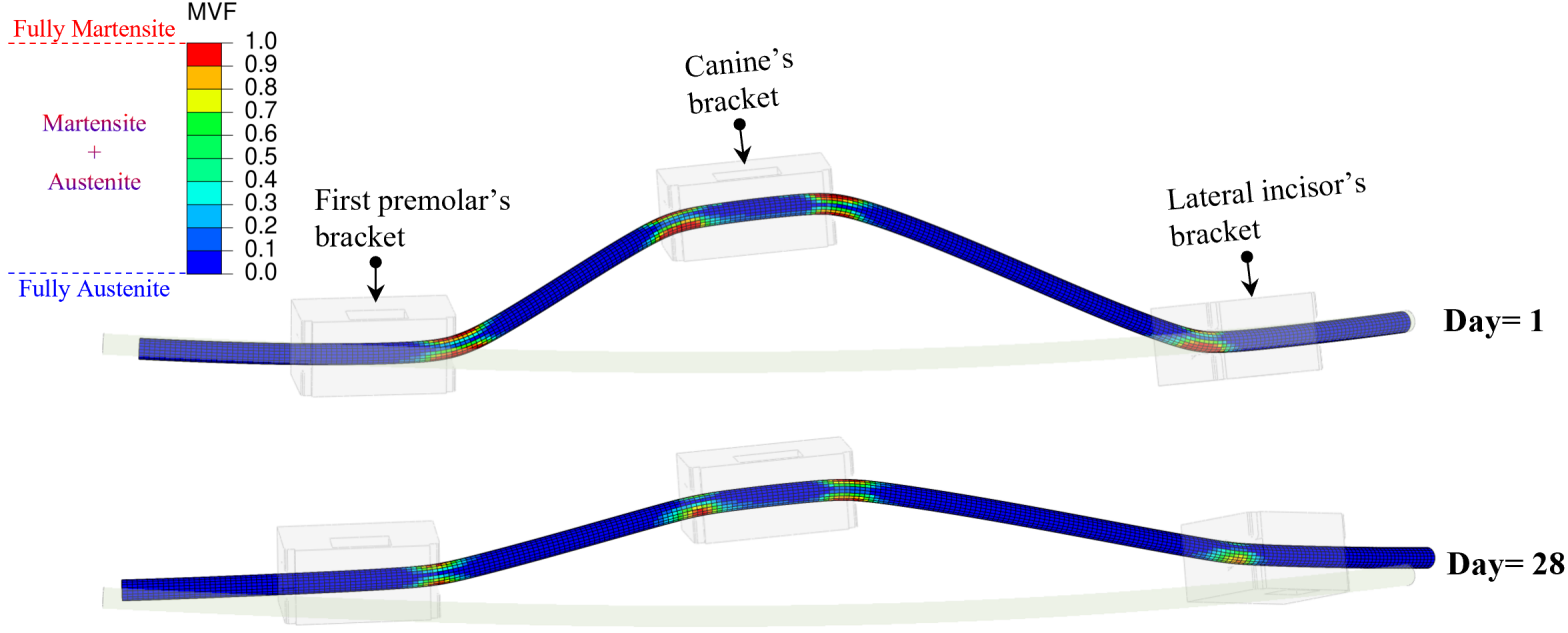
Martensitic volume fraction distribution in the superelastic NiTi arch wire, focusing on the portion that is in contact with the brackets associated with T12, T13, and T14, shown at the beginning and at the end of the treatment period.

Although the model was based on patient‐specific anatomy, it was limited to the movement of a three‐tooth segment while applying a symmetry condition applied at the arch wire midline, the predicted canine displacement over 28 days falls near the lower bound of the wide general clinical OTM range of 0.6–5 mm/month described in the review [44]. However, this range includes different types of OTM, different teeth, heterogeneous malocclusions, and various clinical conditions. For a more case‐specific comparison, the clinically derived traction rate can be estimated from the depth‐dependent guided eruption times reported by Londhe et al. [45] for buccally impacted maxillary canines treated with open or closed surgical exposure followed by light orthodontic traction. For canines located 1–5 mm from the alveolar margin, the reported guided eruption time was approximately 3 ± 1.3 months, corresponding to an estimated traction rate of 0.58–1.16 mm/month. Accordingly, the predicted numerical canine OTM rate remains consistent with clinically reported data for labially/buccally impacted maxillary canine traction.

Beyond this canine‐specific validation, the arch wire applied forces not only to the canine but also to the adjacent lateral incisor and first premolar. These neighboring teeth were consequently displaced in the opposite vertical direction to the canine, contributing to an approximately 37% reduction in the initial vertical gap between the brackets. However, this reduction was accompanied by unwanted intrusion, tipping, and rotational movements of the adjacent teeth. As shown in Figure 8, the teeth were not yet positioned on a common occlusal level, indicating that the leveling phase was not completed with a single initial arch wire. Clinically, this justifies the use of successive arch wire sequences with progressively increasing cross‐sectional dimensions during the alignment phase to gradually align teeth positioned at different vertical levels [46, 47]. Once leveling is achieved, a finishing phase, mainly using rectangular arch wires, is required to correct the unwanted rotations and tipping generated during leveling and to achieve proper root orientation.

This study focused on the simulation of the initial stage of orthodontic alignment using a 0.016‐in. superelastic NiTi arch wire over a one‐month treatment period. Since the ultimate objective of the leveling phase is to obtain a uniform occlusal plane in all three spatial dimensions and given that the literature does not provide a consensual recommendation regarding arch wire sequencing, future work will extend this framework to simulate successive arch wire sequences commonly used in clinical practice. Parametric variations in the bracket type and slot geometry should also be investigated to rationally optimize treatment strategies by maximizing the benefit/risk ratio. In parallel, future development should strengthen the clinical assessment of the proposed approach through longitudinal patient‐specific data. A limitation of the present study is the absence of clinical follow‐up using intraoral scans for direct comparison with the simulated teeth movement. Future work should therefore include prospective tracking of teeth positions in a larger series of clinical cases over defined treatment periods. Such data would allow a quantitative assessment of the model predictions and, if necessary, calibration of the remodeling parameters. In the longer term, this numerical framework could be combined with machine learning approaches to support patient‐specific orthodontic treatment planning and assist clinicians in comparing alternative treatment strategies.

## 5 Conclusions

In this work, a 3D numerical method was developed to simulate a fixed‐appliance orthodontic procedure routinely performed by clinicians to treat malocclusions. The proposed approach enables the quantification of orthodontic loads applied by the arch wire to individual teeth and predicts the resulting alveolar bone remodeling and OTM during the long‐term unloading phase. A clinical case was used to evaluate the predictive capability of the model, in which CBCT scans were employed to reconstruct patient‐specific geometries of the teeth and alveolar bone. The simulated malocclusion corresponded to an infraposition of the maxillary canine, and the analysis focused on the movement of three adjacent teeth in the upper right segment of the dental arch.

The model was simplified by explicitly modeling the periodontal ligaments of the investigated teeth, while the remaining teeth in the segment were considered fixed. An ideal arch form corresponding to a 0.016‐in. superelastic NiTi arch wire was inserted into the slots of the maximum possible number of passive self‐ligating brackets and activated by displacing the non‐engaged brackets. This methodology enabled the investigation of the mechanical response of the NiTi arch wire and its effect on tooth movement and bone remodeling over a 28‐day treatment period, corresponding to the early stage of the leveling phase.

During activation, the bent regions of the wire underwent forward stress‐induced martensitic transformation. During the treatment phase, partial reverse transformation from martensite to austenite allowed the wire to generate light and sustained loads on the three teeth. These orthodontic forces first induced deformation of the PDLs surrounding each tooth root. Subsequent tooth displacement occurred as a consequence of alveolar bone remodeling governed by the pressure‐tension theory, driven by the normal strain distributions within the PDL. The resulting tooth movement patterns were consistent with the clinically reported range in the literature and reflected an early stage of arch alignment, indicating that the leveling phase had not yet been fully achieved with a single initial arch wire.

Despite the inherent challenges associated with developing FE models capable of accurately reproducing clinical orthodontic conditions and capturing both the mechanical action of the arch wire and the bone remodeling process, the proposed automated approach successfully simulated patient‐specific teeth alignment induced by a superelastic NiTi arch wire over the considered treatment period. Future studies will aim to simulate a complete leveling phase using successive shape memory alloy arch wires, while incorporating longitudinal clinical follow‐up data from different malocclusion cases to further assess and improve patient‐specific orthodontic treatment planning.

## Author Contributions

**Mouhamad Abou Hamdan:** writing – original draft, conceptualization, methodology, software, visualization, investigation, formal analysis. **Delphine Wagner:** writing – review and editing, conceptualization, supervision, project administration, validation. **Hamdi Jmal:** writing – review and editing, conceptualization, supervision, project administration, methodology, validation. **Siham Touchal:** writing – review and editing, conceptualization, supervision, project administration, methodology, validation.

## Funding

This work of the Interdisciplinary Thematic Institute HealthTech, as part of the ITI 2021–2028 program of the University of Strasbourg, CNRS and Inserm, was supported by IdEx Unistra (ANR10‐IDEX‐0002) and SFRI (STRAT'US project, ANR‐20‐SFRI‐0012) under the framework of the French Investments for the Future Program.

## Ethics Statement

This study did not involve any clinical experiment, intervention, or prospective recruitment of human participants. Anonymized patient‐specific CBCT data were used only for numerical simulation purposes; ethical approval was not required.

## Conflicts of Interest

The authors declare no conflicts of interest.

## Supporting information


**Video S1:** Profile view of the numerical simulations showing the extrusion of the infrapositioned canine and the intrusion of its adjacent teeth over the 28‐day treatment period. The model features a 0.016‐in. activated NiTi arch wire engaged within the slots of 0.018 × 0.025‐in. passive self‐ligating brackets.

## Data Availability

The authors confirm that all data supporting the findings of this study are included within the article.
